# Parietal Gamma Band Oscillation Induced by Self-Hand Recognition

**DOI:** 10.3390/brainsci12020272

**Published:** 2022-02-16

**Authors:** Masaya Ueda, Keita Ueno, Takashi Inamoto, China Shiroma, Masahiro Hata, Ryouhei Ishii, Yasuo Naito

**Affiliations:** 1 Graduate School of Comprehensive Rehabilitation, Osaka Prefecture University, Osaka 5838555, Japan; ot07092505@gmail.com (M.U.); mc701002@edu.osakafu-u.ac.jp (K.U.); precious.honored.i43.4@gmail.com (T.I.); ishii@psy.med.osaka-u.ac.jp (R.I.); 2 Rehabilitation Unit, Kyoto University Hospital, Kyoto University, Kyoto 6068507, Japan; 3 Department of Comprehensive Rehabilitation, Osaka Prefecture University, Osaka 5838555, Japan; scd03013@edu.osakafu-u.ac.jp; 4 Department of Psychiatry, Graduate School of Medicine, Osaka University, Suita 5650871, Japan; hata_masahiro_mail@yahoo.co.jp

**Keywords:** event-related potential (ERP), time-frequency analysis, self-referential effect

## Abstract

Physiological studies have shown that self-body images receive unique recognition processing in a wide range of brain areas, from the frontal lobe to the parietal-occipital cortex. Event-related potential (ERP) studies have shown that the self-referential effect on the image of a hand increases P300 components, but such studies do not evaluate brain oscillatory activity. In this study, we aimed to discover the self-specific brain electrophysiological activity in relation to hand images. ERPs on the fronto-parietal midline were elicited by a three-stimulus visual oddball task using hand images: the self-hand, another hand (most similar to the self-hand), and another hand (similar to the self-hand). We analyzed ERP waveform and brain oscillatory activity by simple averaging and time-frequency analysis. The simple averaging analysis found no significant differences between the responses for the three stimulus tasks in all time windows. However, time-frequency analysis showed that self-hand stimuli elicited high gamma ERS in 650–900 ms at the Cz electrode compared to other hand stimuli. Our results show that brain activity specific to the self-referential process to the self-hand image was reflected in the long latency gamma band activity in the mid-central region. This high gamma-band activity at the Cz electrode may be similar to the activity of the mirror neuron system, which is involved in hand motion.

## 1. Introduction

The mechanism of self-referential processing is one of the fundamental questions in psychology and neuroscience. The self-referential effects were defined by Rogers et al. [1]. Recently, the pathophysiology of self-referential processing has been studied in patients with autism [2,3], schizophrenia [4,5], and brain damage [6,7]. Body apraxia and body paraphernalia, which occur in people with brain damage, are disorders of body self-awareness [8,9], and means of understanding the mechanisms and developing rehabilitation methods are still under debate [10]. Additionally, it has been shown that self-awareness of the hand and visual factors may affect motor imagery and kinesthetic illusion; this is one of the rehabilitation methods to improve motor function of the upper limbs [11,12,13]. In recent years, there has been interest in body self-awareness mechanisms as self-referential effects, most of which have focused on face recognition processing. These physiological mechanisms were shown by applying electrophysiological methods, such as event-related potential (ERP) measured by electroencephalography (EEG) [14,15,16,17,18,19]. EEG measures brain electromagnetic activity with a high temporal resolution of milliseconds. This property is particularly important for investigating the dynamics of neural activity underlying cognitive processing [20]. However, few studies have provided information on the self-referencing effects of human limbs. In behavioral studies, Frassinetti et al. [21,22] identified a self-advantage effect that led to a faster and more accurate match-to-sample performance for self vs. other body images, such as hands and feet. Additionally, only two EEG studies using the task of discriminating one’s own hand from the hand of another showed the increased P300 and later component amplitude of the ERP in one’s own hand compared to another’s [23,24]. These EEG studies showed interesting temporal features of self-referential processing of brain activity toward hands and suggested that electrophysiological perspectives may be useful to examine the self-referential effects of hands.

There is general agreement that neural networks for the body’s self-awareness include a wide range of brain regions in the midline, the posterior parietal cortex, ventral temporal cortex, anterior insula, and the extrastriate body area, and previous studies applied the functional magnetic resonance imaging (fMRI) technique [25,26,27,28,29]. Although there have been studies on the referential effects of one’s own body parts using EEG with the region of interest as the median, there have been no studies using the brain oscillatory activities called event-related desynchronization (ERD) and synchronization (ERS). ERD/ERS are time-locked components to the event but not phase-locked and can reflect an induced oscillatory response, which cannot be extracted by a simple linear method, such as averaging [30,31]. The brain oscillatory activity associated with the self-referential activity is thought to be alpha-band power [32,33,34,35] and gamma-band power [36,37]. Hence, the time-frequency analysis seems to be appropriate to analyze task-related changes in oscillatory activity or induced response [38].

We hypothesized that the self-hand image elicits specific effects, such as increasing ERP amplitude or differences in induced ERD or ERS. Our main objective was the detection of brain activity specifically evoked by the recognition of one’s own hand. In this study, to minimize the increase in alpha-type error due to multiple comparisons, we recorded EEG from three sensor positions on the scalp at the midline (Fz, Cz, Pz) based on previous studies. In this paper, we examine the self-specific brain electrophysiological activity in response to hand images as self-referential stimuli. We analyzed ERP waveform and brain oscillatory activity elicited by visual images of one’s own or another’s hand using simple averaging and time-frequency analysis.

## 2. Materials and Methods

### 2.1. Subjects

Ten university students (6 male, 4 female; 20–29 years old; average age: 21.3 ± 1.0 years old) gave their informed consent to participate in the research as volunteers. We confirmed by questionnaire that all of them were right-handed, had no medical history of neurological or psychiatric illness, and had normal or corrected-to-normal vision and no visual disturbances, such as color blindness. All subjects had received more than 12 years of education and were free from any drugs or alcohol for at least 72 hours before the test. The explanation on research cooperation was given orally and in writing to all subjects, and they signed an informed consent form. This study was conducted with the approval of the Osaka Prefecture University Graduate School General Rehabilitation Studies Ethics Committee (approval number 2018-201, Osaka, Japan).

### 2.2. External Stimuli

As external stimuli, visual images of the subjects’ hands were individually captured using a digital camera before the experimental setting. An image of each subject’s hand was taken fixed in an intermediate angle position with a black background and around 550-lx illuminance, which was measured using a luminometer. We controlled for ethnic group, age, and gender in subjects. The similarity scores of the hands for each subjects were calculated using PC software (Robust Finder, Canon IT Solutions Co Ltd., Tokyo, Japan). The similarity score was calculated from the normalized correlation to the luminance values of the images. In the case of color images, the luminance values were converted to monochrome images for processing and extraction. Therefore, the difference in luminance values between the model and the target affected the correlation value. Luminance values were greatly affected by hand size and skin color. The image of a hand with the highest similarity score to subjects themselves was labeled as “other1”, and the second-highest as “other2”.

### 2.3. ERP Designs

**Figure 1:**
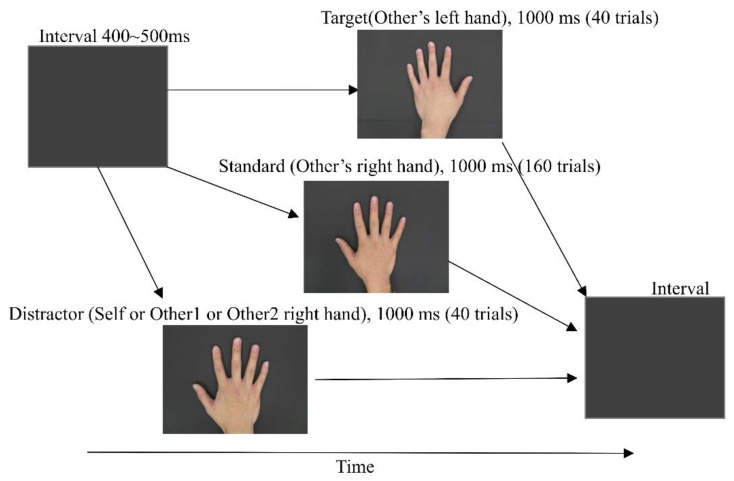
Sample sequence in the visual oddball task.

In this study, we applied three oddball tasks consisting of three visual stimuli to elicit P300 in ERPs to make them reproducible and more reliably represent the differences between oneself and others (Figure 1). Each trial was presented randomly with a black screen as the interval for 400–500 ms. Subjects were instructed to fix their head, minimize eye blinking as much as possible, and push the button by the right hand as soon as possible when the left hand appeared. The total time was about 30 minutes.

All subjects completed the experiments in a practice phase and a test phase. The former phase was to familiarize the subjects with these tasks by having them complete 20 practice trials (15 trials of the right hand and 5 trials of the left hand). The test phase had three experiments (see Figure 1). First, the condition “self” consisted of hand images of oneself or others. The standard stimulus was another’s right hand presented 160 times, the target stimulus was another’s left hand presented 40 times, and the distractor stimulus was one’s own right hand presented 40 times, at random. The stimulation presentation time was 1000 ms, and the interval from the stimulation end to the next stimulation start was set to 400–500 ms. Second, the condition “other1” exchanged the distractor stimulus of the “self” hand to the “other1” hand. Third, the condition “other2” exchanged the distractor stimulus of the “other1” hand to the “other2” hand. Thus, in the three experiments, standard and target stimuli were always the same (another’s left hand and another’s right hand), but the distractor was different (self, other1, other2 right hand). The subjects were instructed to press the button for the target stimulus without giving any information about the visual stimuli of their own hands. The trial order of experiments was completely random. Each task required about 8 minutes.

### 2.4. ERP Recording

The EEG measurement was conducted in a shielded room, and we removed the power line noise by connecting any equipment that could generate alternating currents to the ground. The measured EEG was checked visually, and there was no power line noise. The subjects were in a comfortable position in the seat. The stimulus outputs were displayed using PC software (Stimulus Sequencer, Miyuki Giken, Tokyo, Japan), and the output images were displayed on a 17-inch PC monitor set 60 cm away from the subject’s eyes.

EEG was recorded at 3 sensor positions on the scalp (Fz, Cz, Pz) by using Ag-AgCl dish electrodes (7 mm). The references set the bilateral earlobe attachment sites. The impedance level at the electrodes was set at 10 kΩ or less at all sites. EEGs were recorded without a notch filter. The band-pass filter was from 0.5 to 120 Hz for EEG and EOG, and the sampling rate was 1 kHz.

Electrooculograms (EOGs) were recorded through bipolar leads from the left supra and inferior orbital margins to detect mixed artifacts accompanying blinking and eye movement. To record EEG data, we connected a biosignal recording device (Polymate AP1000, Miyuki Giken Inc., Tokyo, Japan) with a preamplifier (32 Ch electroencephalogram amplifier for Polymate, Miyuki Giken Inc.) with a personal computer (CF-F9 with OS 7, Panasonic, Osaka, Japan). Epochs with artifacts due to eye blink or muscle movements were detected and removed based on their typical signal characteristics and abnormal amplitude information. Only artifact-free epochs were retained for further analysis.

### 2.5. Signal Averaging

The EEG signals for the distractor stimulus from the stimulus presentation start (0 ms) to 1000 ms were averaged and analyzed. P300 components were depicted as the maximum positive potentials observed between 250 and 650 ms after stimulus presentation. Particularly, the amplitude values of typical P3b and early components (P3a) were calculated [39]. EEG analysis software (AP Monitor Version 5, NoruPro Light Systems, Inc. (Tokyo, Japan), Bio Signal Viewer System Version 4, NoruPro Light Systems, Inc. (Tokyo, Japan)) was used for averaging and analyzing ERP. ERP trials with EOG artifacts and bursts of electromyography (EMG) activity (mean EOG and EMG voltage exceeding ± 50 μV) were excluded from further analysis. The pre-stimulus baseline (−100 to 0 ms) was used to perform a baseline correction.

### 2.6. Time-Frequency Analysis

We used the Brain Electrical Source Analysis (BESA) Version 5.0 (BESA GmbH) software to visualize time-frequency representations of the EEG signals in individual subjects. Brain oscillatory activity changes during the perception of each task stimuli of standard, target, and distractor were transformed into the time-frequency domain by using complex demodulation (for detailed information on this methodology, see [40]). To compare each stimulus of each task data, the time and frequency windows for time-frequency analysis were between 0 and 120 Hz and between 0 ms and 1000 ms, respectively. The evoked averaged responses were subtracted from the time series of each trial before the main time-frequency transformation to minimize the contribution of phase-locked components to subsequent estimates of induced activity.

### 2.7. Statistical Analysis

All three electrode sites were selected for statistical analysis (Fz, Cz, Pz). First, we analyzed the 2-way analysis of variance (ANOVA) to the mean amplitudes of ERP for distractor stimuli in the 250–350 ms, 350–500 ms, and 500–650 ms ranges based on research by Su et al. [24]. The factors were condition (self-hand, other1-hand, other2-hand) and electrode sites (Fz, Cz, Pz). The significance level was less than 5%. Using G power 3.1 software, we conducted post-hoc power analyses with an effect size of medium (0.25), an α of 0.05, and a non-sphericity correction ε of 0.7. The correlation among repeated measures for a 2-way ANOVA to the mean amplitudes in the 250–350 ms was 0.23, and the power of analysis was 0.54; for 350–500 ms, the correlation was 0.31, and the power of analysis was 0.59; and for 500–650 ms, the correlation was 0.34, and the power of analysis was 0.61. The power analysis values for these measures were found to be acceptable.

Second, we analyzed time-frequency data averaged for each subject. Statistical analyses were conducted using BESA Statistics 1.0 for permutation testing and cluster analysis. BESA Statistics uses parameter-free permutation testing on the basis of the Student’s *t*-test [41,42]. In this study, there were no predefined clusters, as BESA Statistics 1.0 automatically identifies clusters in time and frequency that are significantly different between 2 conditions. The null hypothesis of “the data under the experimental conditions comes from the same probability distribution” was rejected if at least one t-value was above the critical threshold for *p* < 0.05 determined by 1024 permutation. We compared all subjects’ brain oscillation activities by standard, target, and distractor stimuli within each condition (thus, the comparison was target vs. standard, distractor vs. target, and distractor vs. standard in each condition).

## 3. Results

### 3.1. Simple Averaging Analysis

In all subjects, a clear peak latency of P300 was observed for the target stimuli; however, for the distractor stimulus, it was not found in several subjects. ERP for distractor stimuli, calculated by simple averaging, resulted in large deflections peaking between approximately 250 and 350 ms. Therefore, a mean amplitude of 250–350 ms as a typical P3a component was calculated.

**Figure 2:**
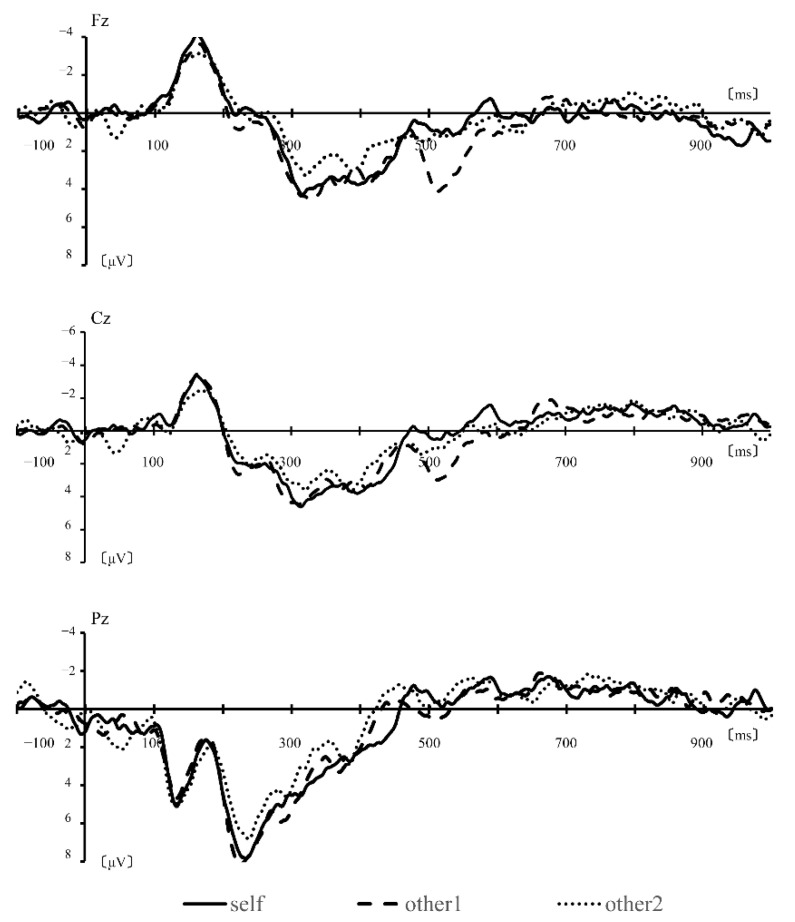
Grand average ERPs from the 10 subjects for each condition.

Figure 2 shows grand mean ERPs for each task distractor stimuli from all scalp EEG channels. In behavioral data, the detection error was lower than 1%, and all subjects pressed the button for target stimuli easily. In all subjects, peak latency of P300 for the target stimuli was clear, but it did not appear in several subjects for the distractor stimulus. Thus, two-way ANOVA was performed to obtain the mean amplitudes of 250–350, 350–500, and 500–650 ms. The results of two-way ANOVA on mean amplitudes of 250–350 ms showed that the interaction between condition and electrode sites was significant [r = 0.23, F (8, 81) = 4.9651, *p* < 0.01], the main effect of each condition was not significant [r = 0.15, F (8, 81) = 2.0367, *p* = 0.1371], and the main effect of electrode site was significant [r = 0.32, F (8, 81) = 10.0952, *p* < 0.01]. On the mean amplitudes of 350–500 ms, the interaction between condition and electrode sites was not significant [r = 0.07, F (8, 81) = 0.487, *p* = 0.7450], the main effect of each condition was not significant [r = 0.08, F (8, 81) = 0.5873, *p* = 0.5582], and the main effect of electrode site was significant [r = 0.19, F (8, 81) = 3.3212, *p* < 0.05]. Mean amplitudes of 500–650 ms showed that the interaction between condition and electrode sites was not significant [r = 0.08, F (8, 81) = 0.5852, *p* = 0.6743], the main effect of each condition was not significant [r = 0.08, F (8, 81) = 0.5753 *p* = 0.5648], and the main effect of electrode site was significant [r = 0.24, F (8, 81) = 5.0590, *p* < 0.01]. In summary, the main effects of electrode sites showed a significant difference in each time window; however, the condition showed no significant difference.

### 3.2. Time-Frequency Analysis

**Figure 3:**
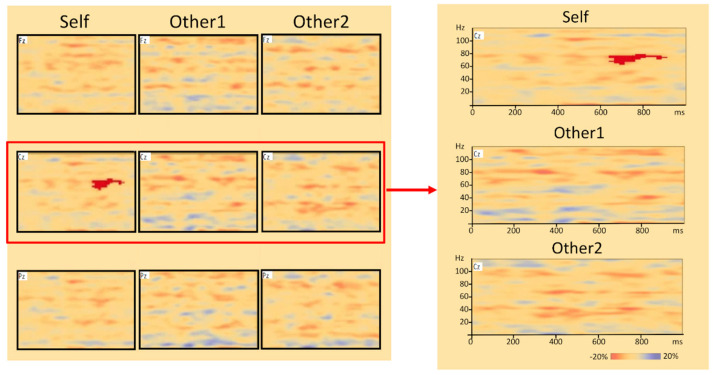
Results of time-frequency analysis. 3 sensor positions on the scalp (Fz = Frontal zero, Cz = Central zero, Pz = Parietal zero) by the international 10–20 system EEG placement were used to record EEG. Paired *t*-test result of distractor–standard stimuli in all subjects and channels of time-frequency data and paired *t*-test result of distractor–standard stimuli only in the Cz channel. Significant increase in high gamma (60–80 Hz) band activity to self-hand was observed within 650–900 ms after stimulus onset in the Cz channel. In the time-frequency plots, the *x*-axis denotes the time relative to the stimulus onset (ms), and the *y*-axis denotes the frequency of oscillatory activity (Hz). The color bar shows the percentage of decrease (blue) and increases (red) in cortical power the 1000 ms post-stimuli.

All electrodes in individual subjects showed a pattern of suppression of oscillatory activity in mu (8–15 Hz) beginning within 200 ms in most stimuli after the stimulus appeared. The result of the time-frequency analysis, the cluster-based permutation test, revealed a significant difference between the distractor stimulus of self-hand and the standard stimulus (*p* < 0.05). Especially, 60–80 Hz frequency (high gamma) band activity in the time range of 650–900 ms at the Cz electrode for distractor stimulus of self-hand was higher than the standard stimuli (Figure 3), whereas there were no significant differences between other1-hand and standard stimuli, and other2-hand and standard stimuli.

## 4. Discussion

In this study, we found that brain cortical oscillatory components for self-hand were significantly larger than for other’s hands in the 60–80 Hz frequency (high gamma) band activity at the Cz electrode in the time range of 650–900 ms. The mean ERP amplitudes obtained using the simple average method were not significantly different between the tasks. These results were consistent with our hypothesis and illustrate that the self-hand image induced a specific late component in the gamma band in the central region.

In our results of the simple averaging analysis, the positive component around 300 ms in Pz was higher than that of the other electrodes. Previous studies that measured ERPs to self-hand reported an increase in P300 at the parieto-occipital electrodes compared to other sites [23,24], and our results follow the previous studies. In contrast, there were no significant differences between all tasks at the ERP amplitude. A direct comparison with previous studies’ results is difficult because these studies used different designs. Our results might be caused by using the visual stimuli of the self-hand without discrimination and the high similarity of the visual stimuli between the self-hand and other hands. The discrimination task used by previous studies may orient attention to one’s own hand because the subjects have no image of other hands. The P300 component usually reflects the course of attention to a stimulus [39,43]. Thus, ERP amplitudes may be susceptible to attention orienteering by tasks and thus may be unsuitable for detecting self-specific responses. However, because the sample size was small, the result of no significant difference between the ERP amplitudes of the self-hand and the other hands should be interpreted with care.

Remarkably, this study revealed a significant difference between the distractor stimulus of self-hand and standard stimulus, and we found that high gamma ERS was induced in 650–900 ms at Cz on self-hand stimuli compared to other hands. ERD/ERS by time-frequency analysis are time-locked components to the event but not phase-locked and can reflect an induced oscillatory response, which cannot be extracted by a simple linear method, such as simple averaging [30,31]. Although there are very few studies measuring evoked oscillatory responses to self-relevant stimuli, Knyazev et al. mentioned the possibility that oscillatory activity specific to self-referencing does occur in the late time window [35]. Our result might have detected self-specific frequency components of brain oscillation activity that are offset as brain noises by simple averaging analysis. Gamma band activity in the neocortex may be generated by responses to sensory stimuli of various modalities and tasks [44,45]. Although the applied time-frequency analysis of hand images as self-referential stimuli has not been reported, several studies reported that the hand motion observation induced high gamma EEG changes [46,47]. Darvas et al. [46] showed the moving hand elicited high gamma activity (70–100 Hz) at the interval from 378 ms to 898 ms around the primary motor area. They suggested that high gamma activity in the observation of biological motion reflects the overall activity of the mirror neuron system. In fact, it has also been reported that high gamma activity at the cortical motor area increased around the hand movement onset and became the most pronounced at the end of the reaching movement [48,49]. Thus, there is a relationship between hand motion and high gamma activity, which can be elicited by observing hand motion. In other words, when motor imagery activity is triggered by visual information of the hand, high gamma band activity associated with hand movement may be observed. In fact, a study using magnetoencephalography to measure brain activity during a hand mental rotation task, which involves motor imagery, gamma band activity in the parieto-occipital lobe was observed [50,51]. Therefore, our results suggest that a visual image of the self-hand may enhance the high gamma activity related to the mirror neuron system, which is supposed to be involved in the hand motion from visual information more than the other hands.

Previous studies have reported that changes in the alpha band activity are mainly associated with self-referential activity [32,33,34,35] and similarly to self-face stimuli [52,53], although similar changes have also been reported when recognizing preferred faces [54,55]. These previous studies may suggest that they roughly reflect top-down processes of visual attention. In this study, top-down process attention was not paid to the self-hand compared with others, as shown by the ERP amplitude results, and therefore, it is possible that a change in the alpha band was not observed.

The fact that the gamma activity in the parietal region band was observed by the self-hand image may support the use of kinesthetic illusion as rehabilitation. It has been reported that kinesthetic illusions could generate motor imagery and might have some effects of restoring motor dysfunction caused by various diseases [56,57] and recovering muscle strength by improving the excitability of the corticospinal tract [58]. However, previous studies have suggested that the effect was different for the image of one’s hand and that of another hand [11,12,13]. Our results suggest the hypothesis that the self-hand is more effective in inducing kinesthetic illusions than other hands.

We should specify some limitations of our research. First, in this study, the sample size was small (10 subjects). Although the statistical power obtained from the two-way analysis of variance for ERP amplitudes calculated using G*power was not small, it may not be sufficient. A concept closely aligned to type II error is statistical power; thus, the result of no significant difference between the ERP amplitudes of the self-hand and the other hands should be interpreted with caution. Second, the spatial resolution of EEG localization was low due to the small number of electrodes used in the measurement. Although we found brain oscillatory responses specific to visual stimuli of the self-hand, the exact localization of this activity was unclear. Further study of where to apply dense electrodes to explore the localization of activity should be conducted. In particular, for the ERPs in the time interval of 150 to 300 ms, the activity at occipital sites could provide valuable information about visual stimuli processing.

## 5. Conclusions

The simple averaging analysis found no significant differences between the responses for three stimulus tasks in all time windows. However, time-frequency analysis showed that self-hand stimuli elicited high gamma ERS in 650–900 ms at the Cz electrode compared with other hand stimuli. The time-frequency analysis might have detected self-specific frequency components of brain oscillation activity offset as brain noises by simple averaging analysis. The visual image of the self-hand may enhance the mirror neuron system related to hand motion more than images of other hands. Our results may bring us some beneficial information for selecting images to facilitate motor function. In future work, we will need to use a dense electrode that provides additional and more exact information.

## Data Availability

All data generated or analyzed during this study are included in this article. Further enquiries can be directed to the corresponding author.
